# ITS rDNA Barcodes Clarify Molecular Diversity of Aquatic Hyphomycetes

**DOI:** 10.3390/microorganisms10081569

**Published:** 2022-08-04

**Authors:** Ricardo Franco-Duarte, Isabel Fernandes, Vladislav Gulis, Fernanda Cássio, Cláudia Pascoal

**Affiliations:** 1 CBMA (Centre of Molecular and Environmental Biology), Department of Biology, University of Minho, 4710-057 Braga, Portugal; 2 Institute of Science and Innovation for Bio-Sustainability (IB-S), University of Minho, 4710-057 Braga, Portugal; 3 Department of Biology, Coastal Carolina University, Conway, SC 29528-6054, USA

**Keywords:** internal transcribed spacer, aquatic fungi, taxonomy, molecular identification, freshwaters

## Abstract

Aquatic hyphomycetes are key microbial decomposers of allochthonous organic matter in freshwater ecosystems. Although their importance in carbon flow and food webs in streams is widely recognized, there are still gaps in our understanding of their molecular diversity and distribution patterns. Our study utilized the growing database of ITS rDNA barcodes of aquatic hyphomycetes (1252 sequences) and aimed to (i) produce new barcodes for some lesser-known taxa; (ii) clarify the taxonomic placement of some taxa at the class or order level, based on molecular data; and (iii) provide insights into the biogeographical origins of some taxa. This study increased the number of aquatic hyphomycete species with available ITS barcodes from 119 (out of ~300 species described) to 136. Phylogenetically, the 136 species were distributed between 2 phyla, 6 classes, and 10 orders of fungi. Future studies should strive to increase the database of ITS sequences, especially focusing on species with unclear phylogenetic relationships (*incertae sedis*) and with few sequences available. The geographical distribution of species with available ITS sequences included 50 countries from five continents, but 6 countries had more than 20 species associated, showing a bias toward the northern hemisphere, likely due to sampling bias.

## 1. Introduction

Aquatic hyphomycetes are the major microbial decomposers of plant litter in streams [1,2]. They play a pivotal role in these ecosystems by driving carbon and nutrient cycling and channeling energy to higher trophic levels [3], thereby contributing to the functioning of freshwater ecosystems. Aquatic hyphomycetes comprise over 300 species of fungi [4,5] with a worldwide distribution [6]. However, the occurrence of individual species is likely to depend on latitude and/or altitude [7,8,9] and to be influenced by physical characteristics of streams and rivers, as well as water chemistry [10,11,12]. A majority of aquatic hyphomycetes belong to the phylum Ascomycota [5,13]. A large number of species are in the class Leotiomycetes, while others are distributed among Sordariomycetes, Dothideomycetes, Orbiliomycetes, and Pezizomycetes [14,15,16,17]. However, the taxonomic positioning of many species of aquatic hyphomycetes remains undefined due to the lack of either teleomorph observations or molecular data.

Conidial morphology still plays a large role in the taxonomy of aquatic hyphomycetes [18,19], with many species producing stauroconidia (mostly tetraradiate spores), variously branched spores, or scolecoconidia (sigmoid, variously curved, or substraight spores). Along with spore shapes, the details of conidiogenesis are also traditionally used in systematics [20,21]. However, the conidial morphologies of aquatic hyphomycetes are believed to have evolved convergently as independent adaptations to similar environmental pressures in different phylogenetic lineages of fungi, making the conidial shape an unreliable indicator of phylogenetic relationships [16]. For some years now, great efforts in fungal taxonomy and systematics have focused on comparisons of nucleotide sequences of select genes instead of, or in addition to, phenotypic characters [22]. DNA sequences are increasingly used to investigate anamorph/teleomorph connections and phylogenetic relationships among fungal taxa [23,24,25,26]. In addition, molecular barcodes, including ITS rDNA sequence data [27], are invaluable in studies dealing with analyses of fungal community structure from environmental samples [9,28,29]. Compared to morphology, molecular data provide considerably more information for phylogenetic analyses and therefore have improved resolving power. For instance, molecular data showed that several genera of aquatic hyphomycetes are polyphyletic [17,18,30], helped to connect anamorphs to teleomorphs [26], and suggested that many aquatic hyphomycetes have relatives of terrestrial origin [18].

In an attempt to better understand phylogenetic relationships among aquatic hyphomycetes, Duarte et al. [31] found that only 26% of all described species had an internal transcribed spacer (ITS) rDNA barcode available. Although there has been an effort to sequence different loci (including ITS) from isolates of aquatic hyphomycetes since then [18,30,32,33], a large-scale comprehensive analysis of available ITS barcodes of aquatic hyphomycetes is still missing. Here, we analyzed sequence data from the ITS rDNA (most widely used DNA barcode for fungi [27]) from all species of aquatic hyphomycetes available in GenBank (119 species) and also included new sequences generated in this study in our labs from 53 pure cultures (41 species, with 17 of them sequenced for the first time). The objectives of our study were to (i) provide new barcodes of aquatic hyphomycetes, (ii) clarify the placement of some taxa at the class- or order-level based on molecular data, and (iii) discuss the biogeographical distribution of aquatic hyphomycetes with ITS barcodes.

## 2. Materials and Methods

### 2.1. Dataset Compilation

We assembled a dataset by compiling available information on aquatic hyphomycetes from the literature and public databases, using as query the terms “aquatic hyphomycetes”, “Ingoldian fungi”, and “Ingoldian hyphomycetes”. Species’ accepted name and synonyms and the information on the teleomorph and anamorph connections from three databases—Mycobank (https://www.mycobank.org/; accessed on 1 January 2021), Index Fungorum (www.indexfungorum.org; accessed on 1 January 2021), and NCBI Taxonomy (https://www.ncbi.nlm.nih.gov/taxonomy; accessed on 1 January 2021)—were compiled (Supplementary Data S1). In addition, ITS rDNA sequences were downloaded from GenBank (www.ncbi.nlm.nih.gov/genbank/; accessed on 1 January 2021), retrieving only sequences that were obtained from pure cultures (metagenomic and environmental studies were not considered).

### 2.2. New ITS Barcodes

Fungi from environmental samples (submerged decaying plant litter or stream foam) were isolated according to Pascoal et al. [34] and Descals [35]. Pure cultures were grown at 15 °C on 1% malt extract agar for approximately 15–20 days before DNA extraction. DNA extractions were performed by using the DNeasy PowerSoil Kit or DNeasy UltraClean Microbial Kit (Qiagen) according to the manufacturer’s instructions. PCR amplifications were performed according to Duarte et al. [36] or Baschien et al. [18], targeting the entire fungal ITS1-5.8S-ITS2 region of the rDNA by using primer pairs ITS1F/ITS4 [37,38] or SR6R/LR1 [39]. PCR products were checked on an agarose gel to confirm the presence of the desired band and then purified by using the PureLink PCR purification Kit (Invitrogen, Walthamm, MA, USA) or ExoSAP-IT PCR product cleanup reagent (Applied Biosystems, Walthamm, MA, USA) according to the manufacturer’s instructions. DNA concentrations were checked with a NanoDrop spectrophotometer (ND-1000, Thermo Fisher Scientific, Walthamm, MA, USA). The amplicons were sequenced in both directions either at StabVida (Oeiras, Portugal) or Eurofins Genomics (Louisville, KY, USA).

Isolates are maintained in culture collections, in particular at the Centre of Molecular and Environmental Biology (CBMA) of the University of Minho, Portugal; at the Czech Collection of Microorganisms (CCM), Czech Republic; and at the Coastal Carolina University, USA. The geographical origin and the substrate of all fungal isolates are given in Table 1.

### 2.3. Phylogenetic Analysis

Consensus sequences of ITS region were obtained with BioEdit software, version 7.2.5 (Hall TA, Raleigh, USA) [40], and were deposited in GenBank, under the accession numbers shown in Table 1. To analyze the phylogenetic relationships of aquatic hyphomycetes, we used the assembled dataset of 1252 sequences and aligned them by using the multiple sequence alignment algorithm FFT-NS2 implemented in MAFFT software version 7 (Katoh K) [41,42]. Maximum likelihood (ML) phylogeny was inferred by using IQ-TREE based on the best-fitting model (SYM + I + G4), according to the Bayesian Information Criterion (BIC), after testing 88 DNA models [43,44], with automated model finder [45] and the bootstrap algorithm UFBoot [46]. Branch support was assessed with bootstrap analysis (1000 replicates) [47]. Dissimilarity between DNA sequences assessed within and between genera was calculated by using MEGA-X software [48]. The ITS sequence of *Mucor hiemalis* (type strain CBS 201.65, GenBank accession number NR_152948.1) was used as the outgroup to root the trees. Phylograms were pruned, formatted, and colored in iTOL [49,50].

## 3. Results and Discussion

We compiled a dataset summarizing data regarding described species of aquatic hyphomycetes (Supplementary Data S1). Our dataset combines taxonomic data for a total of 323 species, in particular, the accepted name, synonyms, basionym, teleomorph, and anamorph, as well as the taxonomic placement (phylum, class, order, and family). The results showed a high taxonomic diversity of aquatic hyphomycetes that were distributed among two phyla (Ascomycota and Basidiomycota), 8 classes, 16 orders, 22 families, and 124 genera. We searched GenBank for the 323 species of aquatic hyphomycetes, and of those, ITS rDNA sequences were found for 119 species and 1073 strains, with a total of 1198 sequences since some strains had more than one sequence deposited, as they were sequenced by different researchers (Supplementary Data S2). Only sequences of aquatic hyphomycetes identified to the species level were considered (i.e., data for specimens identified to the genus level were ignored, e.g., *Flagellospora* sp.). We expanded this database by including new ITS rDNA sequences generated in this study from 53 pure cultures of 41 species of aquatic hyphomycetes, with 17 of them belonging to species for which no ITS data were yet available (Table 1). The 1252 barcodes considered in our study (136 species and 1127 strains) have an average size of 504 base pairs, with some isolates having longer barcodes (up to 975 base pairs), due to the existence of long inserts in the ITS region, especially among Dothideomycetes (data not shown).

Our database represents the largest number of taxa and barcodes compiled so far, much greater than those from other studies, e.g., 19 species and 94 isolates [51]; 7 species and 21 strains [52]; 6 species and 130 isolates [36]; and 75 species [18]. In addition, we generated 53 new ITS barcodes (Table 1). Our results increased the percentage of species of aquatic hyphomycetes with an available ITS barcode from 37% (119 species out of 323) to 42% (136 species out of 323). The latter number still appears to be low, emphasizing the need to generate more barcodes to better understand genetic diversity and to facilitate advances in molecular fungal ecology [53,54]. At the same time, it is likely that some of the 323 species names in our database will be eventually synonymized with others when new molecular evidence becomes available in the future. Thus, we believe that we likely covered at least 50% of the known diversity of aquatic hyphomycetes.

**Figure 1:**
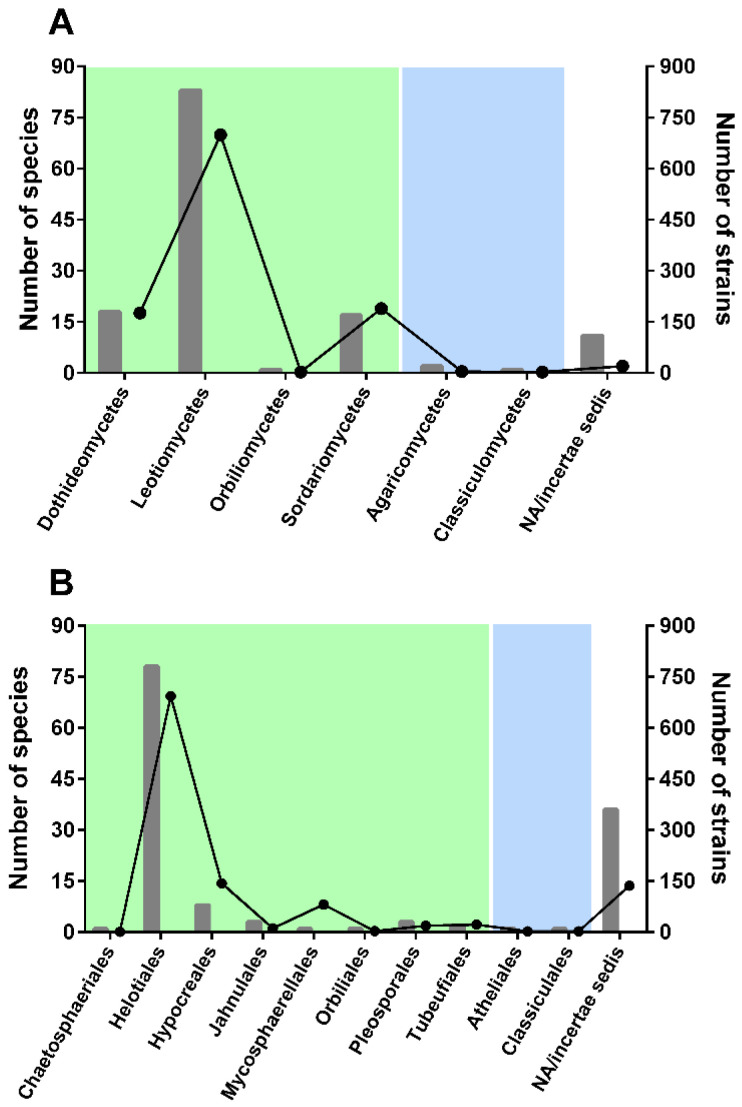
Distribution of aquatic hyphomycete species (bars) and strains (lines) among classes (**A**) and orders (**B**) of ascomycetes (green) and basidiomycetes (blue).

As expected, a much larger number of aquatic hyphomycetes showed an affinity to ascomycetes than to basidiomycetes (Figure 1). 

In Ascomycota, the highest number of species was found to belong to Leotiomycetes (83 species, Figure 1A), with ~700 strains with available ITS sequences (56% of the total sequences considered in our study). Very few species were found to belong to Basidiomycota classes: Agaricomycetes and Classiculomycetes, with only two and one species attributed, respectively. In terms of orders (Figure 1B), Helotiales accounted for the highest number of species and strains—78 and 693, respectively. Notably, a considerable number of species (36 species; 26% of all species considered) could not be attributed to any order (*incertae sedis*). Clearly, additional barcodes should be generated for less represented taxa to better understand genetic diversity and placement of some species.

**Figure 2:**
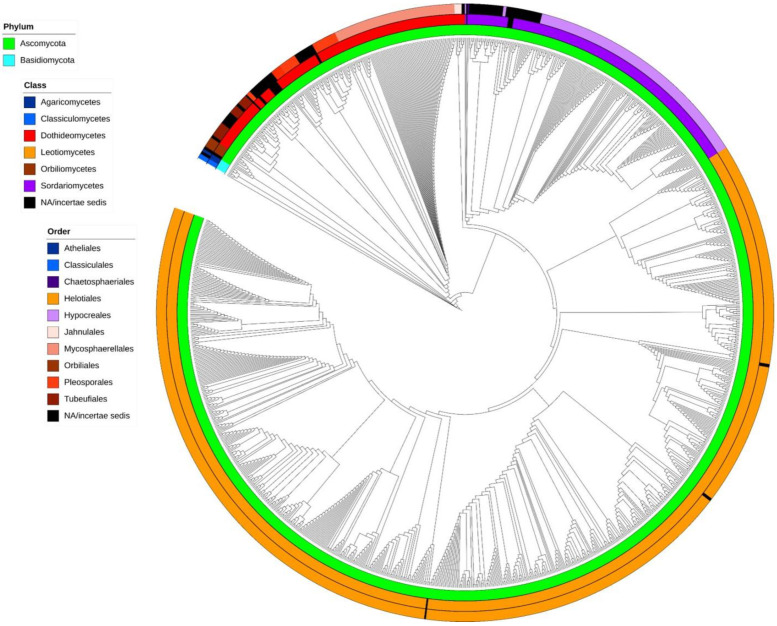
Cladogram showing phylogenetic affinities of aquatic hyphomycetes based on 1252 ITS rDNA barcodes. Circle sections represent taxonomic divisions: inner circle—phyla, middle circle—classes, and outer circle—orders.

A cladogram showing phylogenetic relationships of aquatic hyphomycetes, based on ITS barcodes, is shown in Figure 2 (phylogram in traditional rectangular format is available as Supplementary Data S3). The proposed cladogram divides the 136 species between two phyla, namely Ascomycota (Figure 2, green) and Basidiomycota (Figure 2, blue), and displays the separation of 6 classes and 10 orders into well-defined clades. While the general topology of the tree based on ITS rDNA sequences of aquatic hyphomycetes was reasonable, some species and genera were problematic or not properly resolved. For example, in case of *Tetracladium*, even though the genus represents a well-defined clade, no clear separation among the seven species was evident. Similar results were previously reported by using sequences of 18S rDNA [55], 28S rDNA [56], and ITS+28S [18] regions. The five species of the genus *Lemonniera* also clustered in a well-defined clade, but, as for *Tetracladium*, no clear separation among the species was evident. Interestingly, our results positioned all five species within Leotiomycetes, Helotiales. This contrasts with previous results based on 28S region, where *L. pseudofloscula* was positioned within Dothideomycetes, Pleosporales [16]. Moreover, the genus *Fontanospora* was split into four groups: one group with *F. eccentrica* only; a second with *F. fusiramosa* only; a third group with *F. alternibrachiata*; and a final one with a mix of *F. fusiramosa*, *F. eccentrica*, and *Articulospora tetracladia* (the latter isolate was most probably misidentified, since all the other isolates of *A. tetracladia* clustered together). *Fontanospora* was previously reported to be polyphyletic based on analysis of 28S rDNA [17]. Isolates of *Filosporella versimorpha* (2) and *F. fistucella* (5) are intermingled on a tree, and the same pattern was observed for isolates of *Alatospora flagellata* (2), *A. acuminata* (27), and *Flagellospora leucorhynchos* (1), suggesting that using just ITS rDNA sequences is not sufficient to resolve their phylogenetic relationships. *Anguillospora crassa* separated into two distinct groups, with *Tricladium obesum* and *Anguillospora furtiva* being phylogenetically close; all of these species belong to a recently described family, Tricladiaceae [30]. In our analysis, isolates of *Tumularia aquatica* are separated into two groups within Dothideomycetes. One group clustered with *Colispora cavincola*, *C. elongata*, *Clavariopsis aquatica*, and *Tumularia tuberculata*. The other group (two isolates) formed a separate clade that was distant from the previous one. This may suggest a misidentification of the strains and highlights the importance of using ex-type strains with available DNA barcodes to help in the identification of problematic isolates [57]. Strains identified as *Speiropsis pedatospora* were also separated into two groups. One group (including ex-type culture) was clustered with *Speiropsis scopiformis* within Dothidiomycetes and close to the order Tubeufiales. In a recent study based on ITS + 28S sequences, *S. pedatospora* was positioned in the family Weisneriomycetaceae as a sister group to Tubeufiales [58]. The second group contains likely misidentified isolates (SS2229 and SS2236) and is placed in Jahnulales, Dothideomycetes [59,60,61]. ITS region does not seem suitable to resolve the phylogeny of *Wiesneriomyces laurinus*, since isolates of this species were split into three groups with other species in between (*Speiropsis* spp., *Phalangispora nawawii*, and *P. constricta*). The analysis of both 18S and 28S regions seems to have better resolving power for *Wiesneriomyces laurinus* [62]. We also found problems with a few isolates that did not group together with the remaining isolates of the same species, possibly due to misidentification: *Amniculicola longissima* WPRHD03, *Neonectria lugdunensis* NRRL-20592, *Flagellospora curvula* 30-67, *Anguillospora furtiva* NBRC-103659, *Varicosporium elodeae* AU-CRYP05, and *Articulospora tetracladia* CCM F-12313.

The ITS rDNA region is considered the primary fungal barcode region (e.g., the Consortium for the Barcode of Life 2007 [27,63]), showing better results for species-level identification than other markers [64], and is widely used to analyze the structure of fungal communities from environmental samples [9,28,29]. Even though our study based on ITS barcodes allowed us to clarify the taxonomic placement of many taxa (Figure 2), in line with the previous use in species- or genus-level phylogenies [64,65], results should be treated with caution. Whenever possible, multiple loci (e.g., 28S, beta-tubulin, RPB1, TEF1a, MCM7, etc.) should be used to obtain robust phylogenetic hypotheses including aquatic hyphomycetes, especially considering higher-level taxa. The ITS region, when combined with other molecular markers, showed promising results for taxonomic groups higher than the species or genus level [66,67]. Our results allowed us to shed light on the taxonomic placement of several species whose phylogenetic affinities were previously unclear (black color code in Figure 2). (i) *Campylospora* and *Lunulospora* species are placed within the order Hypocreales in Sordariomycetes (Figure 2 and Supplementary Data S3) with high robustness (bootstraps ≥ 99%). (ii) *Tumularia aquatica* (excluding the two ambiguous sequences) and *Tumularia tuberculata* both group within Dothidiomycetes, and close to species of the order Pleosporales. (iii) Three species (*Goniopila monticola*, *Culicidospora aquatica*, and *C. gravida*) with no clear phylogenetic affinity or with contradictory classification among databases (Mycobank vs. Index Fungorum vs. NCBI) were positioned within the order Helotiales in Leotiomycetes. *C. gravida* was attributed to Helotiales based on the ITS barcode produced for the first time in our study. More sequences from *C. aquatica* and *C. gravida* are needed to increase robustness of these observations. (iv) *Speiropsis scopiformis*, *Phalangispora nawawii*, and *Phalangispora constricta* are within Dothidiomycetes, but further analyses are needed to define their position in terms of order. Other species were attributed to classes, but because only one sequence of each species is available, these observations need to be confirmed in the future. (v) *Retiarius bovicornutus* and *Isthmotricladia gombakiensis* are clustered close to *Dactylellina appendiculata* within Orbiliomycetes. (vi) *Heliscella stellata* is close to *Stenocladiella neglecta* and *Isthmomyces lanceatus* in Dothidiomycetes. (vii) *Lateriramulosa uni-inflata*, *Colispora cavincola*, and *C. elongata* are also placed in Dothidiomycetes.

The highest average evolutionary divergence for all ITS sequences of aquatic hyphomycetes was found between genera *Stenocladiella* and *Classicula* (0.647; Supplementary Data S4). Actually, *S. neglecta* is an ascomycete (Dothidiomycetes) while *C. sinensis* is a basidiomycete (Classiculomycetes). Regarding the average evolutionary divergence within genus (between two or more species of the same genus), the highest divergence was observed for *Mycofalcella* (0.22) and the lowest for *Aquanectria* (0.0081) and *Variocladium* (0.0085). The genus *Mycofalcella* comprises two species: *M. calcarata*, which was recently repositioned in the family Tricladiaceae (Helotiales, Leotiomycetes) [30], and *M. iqbalii*, which is also connected to Tricladiaceae (Helotiales, Leotiomycetes), according to Mycobank and Index Fungorum. However, in our analysis, *M. iqbalii* is positioned within Dothideomycetes, which explains the high estimate of evolutionary divergence. Future analysis using additional markers is needed to confirm the position of *M. iqbalii*. *Aquanectria* (Hypocreales, Sordariomycetes) is a recent genus erected to accommodate two species (*A. penicillioides* and *A. submersa*) previously in the genera *Flagellospora* (as *F. penicillioides*) and *Heliscus* (as *H. submersus*), respectively [68]. The genus now includes five more species described based on multilocus phylogenetic analyses [69,70], but none of the new species is considered to be aquatic hyphomycetes. *Variocladium* contains two species, *V. giganteum* and *V. rangiferinum* (Helotiales, Leotiomycetes) [17,71]. Interestingly, the average evolutionary divergence within genera *Aquanectria* (0.0081) and *Variocladium* (0.0085) was lower than that of the species *Vibrissea flavovirens* (0.0086) (Supplementary Data S4). Thus, *Aquanectria* and *Variocladium* illustrate a situation of quick morphological diversification and/or slow molecular evolution of ITS region.

In our study, 46 species were represented by just a single sequence each. This may influence conclusions about the taxonomic placement of these particular species due to possible misidentification of isolates or inaccurate sequences deposited in public collections. We encourage continuous efforts to isolate and produce new DNA barcodes of aquatic fungi. In addition, a larger number of DNA sequences from strains isolated from different geographical regions are needed to provide an opportunity to address other questions such as haplotype analysis [72,73] in aquatic hyphomycetes.

**Figure 3:**
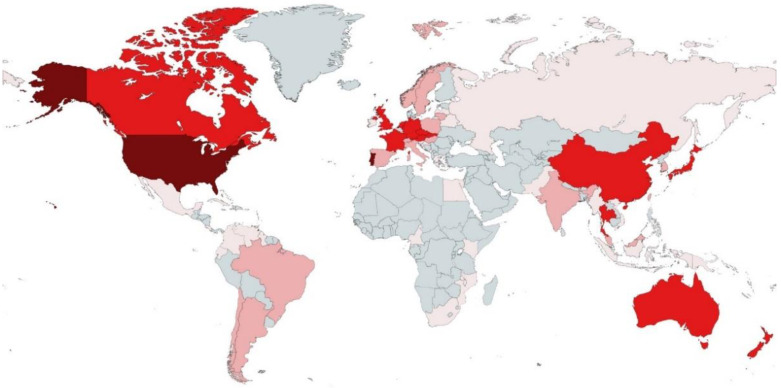
World map showing the countries of origin for strains/sequences of aquatic hyphomycetes used in this study. Colors indicate the number of different species obtained from each country: 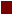
—more than 30; 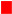
—from 11 to 29; 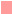
—from 3 to 10; 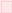
—less than 3 species; 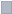
—zero species.

The geographical origin of all species with available ITS sequences included a total of 50 countries from five continents (Figure 3 and Supplementary Data S2).

North American and European countries had the highest number of species of aquatic hyphomycetes with available ITS barcodes: Portugal (34 species), United States of America (33 species), United Kingdom (29 species), Germany (28 species), France (22 species), and Czech Republic (21 species). These findings suggest that a larger effort isolating and barcoding aquatic hyphomycetes is still needed, especially in some parts of the world without any representation, such as in many countries of Africa and some in Asia. Clearly, the number of species reported here for different countries does not necessarily indicate the level of biodiversity but rather reflects collecting efforts or the existing expertise in the individual countries.

## 4. Conclusions

This study used the largest dataset of ITS rDNA barcodes (1252) of aquatic hyphomycetes to advance our understanding of phylogenetic relationships among these fungi and their biogeographical origins. Our data showed that 136 species of aquatic hyphomycetes were distributed between the fungal phyla Ascomycota and Basidiomycota, in 6 classes and 10 orders. We generated new barcodes for 17 species and clarified the taxonomic positions of some genera and species, which were previously classified as *incertae sedis*. Future studies should strive to increase the database of ITS sequences, especially focusing on species with unclear phylogenetic relationships (*incertae sedis*). A greater effort in regard to sampling, isolating, and sequencing aquatic hyphomycetes from geographically less explored regions is crucial, particularly from Africa and certain Asian countries. It would also be useful to explore extreme habitats (e.g., intermittent streams, polar regions, and deserts). In addition to sequencing rDNA loci, a multilocus approach including structural gene analysis or comparison of entire genomes might help to provide new insights into fungal classification [22,74]. Whole-genome sequencing and annotation will also facilitate the study of the phylogeography of aquatic hyphomycetes, while environmental metagenomics will help to unravel patterns of their distribution in aquatic ecosystems, including those affected by anthropogenic impacts. These modern approaches that allow for species detection in the absence of reproductive structures may open new avenues to fungal conservation [75].

## Figures and Tables

**Table 1:** Sources and accession numbers of aquatic hyphomycete isolates sequenced in this study. Species with new ITS barcodes generated for the first time in this study are highlighted in bold.

Species Name	Strain	Isolation Country	Isolation Substrate	GenBank Accession Number
*Alatospora acuminata* Ingold	UMB-223	Portugal	foam	OM273714
*Alatospora acuminata* Ingold	UMB-741	Portugal	leaves	MZ773535
*Alatospora acuminata* Ingold	UMB-902	Portugal	oak leaves	OM273715
*Alatospora pulchella* Marvanová	UMB-1115	Portugal	oak leaves	MZ773536
*Anguillospora crassa* Ingold	UMB-217	Portugal	foam	OM273716
*Anguillospora crassa* Ingold	UMB-1150	Portugal	foam	MZ773539
*Anguillospora crassa* Ingold	VG33-1	USA	dead submerged tree roots	OM907724
*Anguillospora curvula* S.H. Iqbal	VG69-4	USA	grass blades	OM907725
*Anguillospora filiformis* Greath.	UMB-016	Portugal	leaves	OM273717
*Anguillospora filiformis* Greath.	UMB-225	Portugal	leaves	MZ773533
*Aquanectria penicillioides* (Ingold) L. Lombard and Crous	VG205-1-2	USA	wood	OM907726
***Arbusculina irregularis*** (R.H. Petersen) Marvanová and Descals	**CCM F-23687**	Canada	unknown	OM273718
***Arbusculina irregularis*** (R.H. Petersen) Marvanová and Descals	**VG76-8**	USA	foam	OM906795
*Articulospora atra* Descals	VG233-6	USA	wood	OM907727
*Articulospora proliferata* A. Roldán and W.J.J. van der Merwe	VG229-6	USA	grasses	OM907728
*Articulospora tetracladia* Ingold	UMB-712	Portugal	foam	OK605572
*Articulospora tetracladia* Ingold	UMB-1144	Portugal	foam	OK605573
***Casaresia sphagnorum*** Gonz. Frag.	**VG7-1**	USA	*Quercus prinus* leaves	OM907729
***Clavariana aquatica*** Nawawi	**VG75-4**	USA	foam	OM907730
*Clavatospora longibrachiata* (Ingold) Sv. Nilsson ex Marvanová and Sv. Nilsson	VG80-6	USA	*Tilia* sp. leaves	OM907731
***Culicidospora gravida*** R.H. Petersen	**VG39-4**	USA	foam	OM907732
***Dendrosporomyces prolifer*** Nawawi, J. Webster and R.A. Davey	**VG258-1**	USA	foam	OM906797
***Dendrosporomyces prolifer*** Nawawi, J. Webster and R.A. Davey	**VG98-3**	USA	foam	OM906796
*Dimorphospora foliicola* Tubaki	UMB-215	Portugal	leaves	OM273719
*Dimorphospora foliicola* Tubaki	UMB-1119	Portugal	oak leaves	MZ773538
*Filosporella exilis* Gulis and Marvanová	VG211-1	USA	grasses	OM907733
*Filosporella fistucella* Marvanová and P.J. Fisher	UMB-007	Portugal	water	OM273720
***Fontanospora alternibrachiata*** Dyko	**VG8-4**	USA	*Rhododendron maximum* leaves	OM907734
***Geniculospora inflata*** (Ingold) Sv. Nilsson ex Marvanová and Sv. Nilsson	**VG79-1**	USA	twigs	OM907735
***Heliscella stellata*** (Ingold and V.J. Cox) Marvanová	**VG254-5**	S. Korea	*Betula* sp. leaves	OM907736
***Heliscina antennata*** Marvanová	**VG50-2**	USA	artificial foam	OM907737
*Hydrocina chaetocladia* Scheuer	UMB-1116	Portugal	oak leaves	MZ773531
***Isthmotricladia gombakiensis*** Nawawi	**VG113-5**	USA	foam	OM907738
***Lateriramulosa uni-inflata*** Matsush.	**VG80-7**	USA	unident. dicot leaves	OM907739
*Lemonniera alabamensis* R.C. Sinclair and Morgan-Jones	UMB-594	Portugal	leaves	MZ773530
*Lemonniera aquatica* De Wild.	VG66-7	USA	sedges	OM907740
*Lemonniera cornuta* Ranzoni	VG77-4	USA	foam	OM907741
***Lemonniera pseudofloscula*** Dyko	**VG30-2**	USA	*Acer rubrum* leaves	OM907742
*Lemonniera terrestris* Tubaki	VG209-3	USA	leaves	OM907743
*Mycofalcella calcarata* Marvanová, Khattab and J. Webster	VG44-4	USA	decorticated branch	OM907744
*Neonectria lugdunensis* (Sacc. and Therry) L. Lombard and Crous	UMB-161	Portugal	Twigs	OK605576
***Pleuropedium multiseptatum*** Marvanová and Descals	**CCM F-46594**	Canada	Unknown	OM273721
***Pyramidospora constricta*** N. Singh	**VG116-5**	USA	*Platanus* sp. leaves	OM907745
***Pyramidospora ramificata*** Miura	**VG54-1**	USA	unident. dicot leaves	OM907746
***Tricladium curvisporum*** Descals	**VG69-3**	USA	Grasses	OM907747
***Tricladium curvisporum*** Descals	**VG242-1**	USA	Grasses	OM907748
*Tricladium splendens* Ingold	UMB-414	Portugal	Foam	OK605580
*Tricladium splendens* Ingold	UMB-1117	Portugal	oak leaves	MZ773537
*Tumularia tuberculata* (Gönczöl) Descals and Marvanová	VG262-4	S. Korea	*Quercus* sp. leaves	OM907749
*Tumularia tuberculata* (Gönczöl) Descals and Marvanová	VG264-4	S. Korea	*Quercus* sp. leaves	OM907750
*Varicosporium elodeae* W. Kegel	UMB-878	Portugal	Foam	OK605582
*Variocladium giganteum* (S.H. Iqbal) Descals and Marvanová	VG43-4	USA	*Quercus* sp. leaves	OM907751
***Variocladium rangiferinum*** (Descals) Descals and Marvanová	**VG71-1**	USA	Sedges	OM907752

## Data Availability

Accession numbers for DNA sequences obtained for the first time in this study are listed in Table 1. Accession numbers for DNA sequences obtained from GenBank are listed in Supplementary Data S2.

## Supplementary Materials

The following supporting information can be downloaded at https://www.mdpi.com/article/10.3390/microorganisms10081569/s1. Supplementary Data S1: List of species of aquatic hyphomycetes. Supplementary Data S2: Species of aquatic hyphomycetes with available ITS rDNA barcodes considered in this study. Supplementary Data S3: Phylogram of aquatic hyphomycetes based on 1252 ITS rDNA barcodes. Supplementary Data S4: Evolutionary divergence within and between genera.
