# Supplementary material for: ITS rDNA Barcodes Clarify Molecular Diversity of Aquatic Hyphomycetes
Source: Microorganisms. 2022 Aug 4;10(8):1569. doi: 10.3390/microorganisms10081569 (PMC9415940; doi:10.3390/microorganisms10081569)

Supplementary Data S3: Phylogram of aquatic hyphomycetes based on 1248 ITS rDNA barcodes

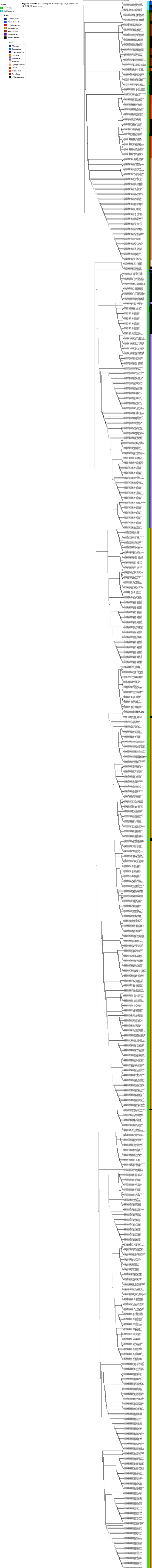

Supplement: Supplementary file 1 [file Supplementary Data S3.pdf › Supplementary Data S3.pdf]
